# A mechanism for reversible mesoscopic aggregation in liquid solutions

**DOI:** 10.1038/s41467-019-10270-5

**Published:** 2019-05-30

**Authors:** Ho Yin Chan, Vassiliy Lubchenko

**Affiliations:** 1 Department of Chemistry, University of Houston, Houston, TX 77204-5003 USA; 2 Department of Physics, University of Houston, Houston, TX 77204-5005 USA

**Keywords:** Computational biophysics, Coarse-grained models

## Abstract

Solutions of proteins and other molecules exhibit puzzling, mesoscopically sized inclusions of a solute-rich liquid, well outside the region of stability of the solute-rich phase. This mesoscopic size is in conflict with existing views on heterophase fluctuations. Here we systematically work out a microscopic mechanism by which a metastable solute-rich phase can readily nucleate in a liquid solution. A requisite component of the mechanism is that the solute form long-lived complexes with itself or other molecules. After nucleated in this non-classical fashion, individual droplets grow until becoming mechanically unstable because of a concomitant drop in the internal pressure, the drop caused by the metastability of the solute-rich phase. The ensemble of the droplets is steady-state. In a freshly prepared solution, the ensemble is predicted to evolve in a way similar to the conventional Ostwald ripening, during which larger droplets grow at the expense of smaller droplets.

## Abstract (web summary)

Solutions of proteins and other molecules can host puzzling, solute-rich inclusions of mesoscopic dimensions. Here the authors report a mechanism by which mesoscopic clusters can nucleate and ripen, requiring that the solute form long-lived complexes, with implications for biologically and industrially relevant systems.

## Introduction

Spatially and chemically heterogeneous systems are of prime significance in the context of both man-made processes, such as self-assembly and nano-particle manufacturing, and naturally occurring systems, such as membrane-less organelles^1–4^. Heterophase inhomoneneities, such as those arising during spinodal decomposition, represent an important subclass of those phenomena. They however become increasingly difficult to produce away from the conditions for inter-phase equilibrium^5^. If appeared, domains of a metastable phase will promptly evaporate because nucleation of such a phase is an uphill process, free energy-wise, owing both to the bulk free energy cost and the mismatch penalty between the majority and minority phase^6,7^. Thus in equilibrium, one has essentially a dichotomy: heterophase inhomogeneities are either of macroscopic dimensions, during a phase coexistence, or, otherwise, could survive only on molecular timescales implying correspondingly small lengthscales.

It then comes as a surprise that equilibrated solutions of several proteins must host mesoscopically sized inclusions of what seems to be a distinct, protein-rich phase of fluid consistency^8–18^; these inclusions are often called “the mesoscopic clusters.” Cluster-containing solutions are stable on time scales of a few months^12^. In systems studied so far, the mesoscopic clusters contain a small fraction of the solute, less than 10^−3^, and thus do not affect the appearance of the solution; common methods of detection include dynamic light scattering, direct tracking using fluorescence, and also atomic force microscopy. In addition to solutions of many proteins, mesoscopic clusters have been recently observed in solutions of relatively simple molecules, viz., the pharmaceutical olanzapine^19^.

The mesoscopic clusters are important for many reasons: They serve as essential nucleation sites for solid protein aggregates such as fibers of sickle cell anemia and tumor suppressor p53^18,20,21^ and protein crystals^8,14,17,22^. Thus by deliberately inducing the formation of clusters, one can seed formation of solid aggregates of interest in applications. Equally important is that the clusters form an ensemble of objects whose size is narrowly distributed around a steady-state value. This may provide a separate avenue for making mesoscopically sized particles or gels in industrially relevant quantities. On the more fundamental side, the existence of mesoscopic clusters suggests a tantalizing possibility that the precursors to living cells were not encased in membranes but, instead, were more like the so called membrane-less organelles. Differing from the surrounding cytoplasm chemically, membrane-less organelles^1–4^ essentially serve as cell’s chemical reactors; the lack of a membrane provides for ready exchange of reactants and products with the cytoplasm. In view of the continuously growing number of cluster sightings, it stands to reason that the clusters are more common than one might think, but are not detected more frequently either because they are a kinetic intermediate to a more stable phase or simply for the lack of trying.

The mesoscopic clusters are not micelle^23^-like objects. This is evidenced by the fact that the mole fraction of the clusters increases gradually with the concentration of the solute, the latter concentration showing no saturation; the value of the mole fraction is consistent with estimates of the free energy cost of creating bulk solute-rich liquid^11^. At the same time, the typical size of an individual cluster does not sensitively depend on the solute concentration. This is in contradistinction to macroscopic phases, which respond to changing conditions by evolving in size until the solution is again saturated. Still in one particular way the clusters in freshly prepared solutions behave similarly to macroscopic phases: Well before its steady-state value is reached, the typical cluster size depends on time^12^ in a way reminiscent of Ostwald ripening^24–27^.

The lack of dependence of the steady-state cluster size on the solute concentration in the bulk solution suggests an additional, molecular-level process is at work. Pan et al.^11^ proposed that this additional process involves the formation and decay of a solute-containing species, call it the “complex.” The complex could contain one or more solute molecules. In this mechanism, the solute-rich phase is assumed to be rich in the complex. The complex would have to have a relatively long lifetime—of the order milliseconds for protein solutions. The cluster size *R* is essentially determined by the distance the complex can diffuse before it decays:

1 $$R \approx \sqrt {D_c/k} .$$

where *k* is the decay rate of the complex and *D*_*c*_ its diffusivity. The lengthscale *R* emerges self-consistently as a result of solving a set of reaction-diffusion equations applicable outside the cluster^11^. Inside the cluster, the equations become however invalid and, furthermore, produce unphysical singularities. A good deal of indirect experimental evidence exists for the “complexation” scenario^11,28,29^, direct observation of the complexes complicated by the small volume fraction of the clusters.

Lutsko and Nicolis^30^ (LN) extended the Pan et al.’s treatment to explicitly include particle-particle interactions using a standard approximation of the theory of liquids. These authors concluded that the resulting reaction-diffusion equations allow for a stationary solution in the form of stable individual clusters, a startling result indeed. Note such a stationary solution would not allow for Ostwald-like ripening but, instead, would exhibit simpler, exponential kinetics for the relaxation of cluster size. At the same time, the only known mechanism for bona fide Ostwald ripening—which may or may not apply to the clusters—requires that droplets surrounded by under-saturated solution evaporate while droplets surrounded by over-saturated solution grow indefinitely^24,25^. Perhaps fittingly, Lutsko^31^ concluded in a subsequent analysis that realistically accounting for the variability of the kinetics depending on the solute concentration would disrupt the complexation mechanism put forth in ref. ^11^, after all.

Here we present a reaction-diffusion treatment applicable throughout the whole space. It demonstrates that the complexation scenario can, in fact, lead to the emergence of a metastable minority phase that is fragmented into inclusions of substantial yet non-macroscopic size, or “clusters.” In contrast with the conclusions of the LN study, individual clusters are never stable in the present mechanism. Once nucleated, the clusters grow precipitously until they become mechanically unstable and break apart via necking. Thus in an equilibrated solution, the droplets nucleate, grow, and decay at a steady rate leading to a steady-state ensemble of clusters, each of which is not steady-state individually. The probability distribution for the cluster size can be centered, within a broad parameter range, at a much larger size than that suggested by the standard theory of heterophase fluctuations^5,11^.

The formation of the transient complexes serves to effectively provide partial, kinetic stabilization of the minority phase but on lengthscales comparable to the distance a complex can travel before it decays; the complexation mechanism requires that the solute-rich phase be rich in the complex as in ref. ^11^. Thus the question of whether the clusters could nucleate is the question of whether microscopic parameters could conspire to make the critical size for cluster nucleation shorter than the kinetic length from Eq. (1). Here we show that, indeed, there is a substantial range of microscopic parameters for which the answer to this question is affirmative. At the same time, the cluster size at the mechanical stability edge does not change much within that parameter range, which is consistent with the observed behavior in protein solutions. Non-withstanding the kinetic character of the effective bulk stabilization of the minority phase, due to the complex formation, cluster nucleation shares an important aspect with nucleation of a stable minority phase: The effective value of saturated vapor pressure still depends on the cluster size. Thus one expects the clusters should exhibit Ostwald-like ripening at sufficiently early times, again consistent with observation. Finally, many types of solutes exhibit a propensity for the formation of transient complexes, even if short-lived. Thus we predict mesoscopic clusters should be observed commonly even if not universally.

## Results

### Setup of the calculation

For concreteness, we assume that the solute-containing complex is a dimer. The coordinate-dependent concentrations of the solute (“the monomer”) and the complex (“the dimer”) are denoted with *n*_1_ and *n*_2_, respectively. The corresponding reaction-diffusion scheme is

2 $$\begin{array}{*{20}{l}} {\dot n_1} \hfill & = \hfill & { - {\bf{\nabla }}{\mathbf{j}}_1 - k_1n_1^2 + 2k_2n_2} \hfill \\ {\dot n_2} \hfill & = \hfill & { - {\bf{\nabla }}{\mathbf{j}}_2 + \frac{1}{2}k_1n_1^2 - k_2n_2,} \hfill \end{array}$$

where **j**_*i*_ is the flux of species *i*, *k*_1_ the (bi-molecular) rate of binding of the monomer to itself, and *k*_2_ the dissociation rate of the dimer. Strictly speaking, the reaction terms in Eq. (2) should be written using the activities, not concentrations; we will return to this notion shortly. In addition, these equations must properly contain terms that account for thermal noise. The latter terms are not included in the treatment since we are mostly interested in the relaxation of the system toward its steady state.

The transport for each species is overdamped at the conditions of interest and thus obeys the usual Fick’s law^26^:

3 $${\mathbf{j}}_i = - \tilde D_i{\bf{\nabla }}\mu _i,$$

where $\tilde D_i$ is the self-diffusivity of species *i* and *μ*_*i*_ its chemical potential. To include off-equilibrium situations in the treatment, we allow both the chemical potentials and concentrations of the monomer and dimer to be coordinate-dependent. The local value of the chemical potential, by construction, is the free energy cost of adding a particle to the system at the locale in question:

4 $$\mu _i({\mathbf{r}}) = \frac{{\delta F}}{{\delta n_i({\mathbf{r}})}},$$

where *F* is the total free energy of the system and *δ*/*δn*_*i*_ is the functional derivative with respect to *n*_*i*_^26,32,33^.

It is guaranteed^34^ that there is a unique free energy density functional that is optimized by the equilibrium density profiles. Aside from the use of concentrations in place of activities, Eqs. (2)–(4) provide an internally consistent, complete description of transport and inter-conversion of the monomer and dimer.

As a practical matter, one uses an approximate form for the free energy functional such as the venerable Landau-Ginzburg-Cahn-Hilliard^35^ functional, which we employ here as well:

5 $$F = {\int} {\left[ {\frac{{\kappa _1}}{2}\left( {\nabla n_1} \right)^2 + \frac{{\kappa _2}}{2}\left( {\nabla n_2} \right)^2 + {\cal{V}}\left( {n_1,n_2} \right)} \right]} d^3{\mathbf{r}}.$$

The latter functional affords one a quantitative description not too close to criticality^32^. The quantities *κ*_*i*_ are the standard coefficients at the square gradient of the order parameter in the Landau–Ginzburg functional^32^ and reflect the free energy cost of spatial inhomogeneity in the order parameter. We assume that the monomer-dimer-buffer mixture can have two distinct liquid phases, one monomer-rich and the other dimer-rich. The bulk portion of the corresponding free energy functional, ${\cal{V}}\left( {n_1,n_2} \right)$, thus has two distinct minima, which makes the solution of Eqs. (2)–(4) difficult in the interfacial region. These difficulties can be efficiently addressed^36^, as we detail in the Methods, by adopting parabolic free energy profiles everywhere within individual phases. Explicit examples of crossing, nearly-parabolic free energy surfaces are provided by Talanquer^37^, who discusses phase behavior of self-associating fluids; the present setup can be viewed as a limiting case of that study where the oligomers do not exceed 2 in size. Thus the bulk free energy of the mixture is set, by construction, at

6 $${\cal{V}}\left( {n_1,n_2} \right) = \mathop {{\min }}\limits_\alpha \left[ {g^{(\alpha )} + \frac{{m_1^{(\alpha )}}}{2}\left( {n_1 - n_{1,b}^{(\alpha )}} \right)^2 + \frac{{m_2^{(\alpha )}}}{2}\left( {n_2 - n_{2,b}^{(\alpha )}} \right)^2} \right],$$

where *α* labels the phase: *α* = m for the monomer-rich, and *α* = d for the dimer-rich solution. The quantity $n_{i,b}^{(\alpha )}$ denotes the equilibrium bulk value of the concentration of species *i* in phase *α*. These are connected with the rate constants according to $k_1^{(\alpha )}(n_{1,b}^{(\alpha )})^2 = 2k_2^{(\alpha )}n_{2,b}^{(\alpha )}$. The coefficients $m_i^{(\alpha )}$ reflect the free energy penalty for density fluctuations and are proportional to the pertinent inverse osmotic compressibility.

In the present treatment of thermodynamics (*κ*_*i*_, *m*_*i*_), transport $(\tilde D_i)$, and chemical transformation (*k*_*i*_), we are performing a quadratic expansion around the bulk equilibrium state for each individual phase. This amounts to our effective use of concentration-independent coefficients *κ*_*i*_, diffusivities $\tilde D_i$, and rate coefficients *k*_*i*_, while writing down the kinetic terms in Eq. (2) in terms of concentrations, not activities. Assumed to be constant within individual phases, these coefficients generally differ between distinct phases. Clearly, the variation of the parameters between the phases, not within individual phases, is the most important effect. The present approach captures this effect. We note that the four diffusivities—there are two species and two phases—are not independent. For internal consistency, one must set $\tilde D_1^{({\mathrm{m}})}/\tilde D_2^{({\mathrm{m}})} = \tilde D_1^{({\mathrm{d}})}/\tilde D_2^{({\mathrm{d}})}$, see Methods for details.

Additional computational difficulties are caused by the presence of the non-linear term $k_2n_1^2$ in Eq. (2). We have numerically solved the resulting non-linear differential equations for several realizations of parameters—to be discussed in due time—however the majority of the calculations were performed for a linearized version of Eq. (2) so that the interconversion between the two species is effectively a first order reaction:

7 $$\begin{array}{*{20}{l}} {\dot n_1} \hfill & = \hfill & { - {\bf{\nabla }}{\mathbf{j}}_1 - k_1n_1 + k_2n_2} \hfill \\ {\dot n_2} \hfill & = \hfill & { - {\bf{\nabla }}{\mathbf{j}}_2 + k_1n_1 - k_2n_2,} \hfill \end{array}$$

where $k_1n_{1,b} = k_2n_{2,b}$ in each phase. Note that if one considers Eq. (7) as a linearized version of Eq. (2), a variable change 2*n*_2_ → *n*_2_ is implied. Equations (7) can also be considered on their own merit: They can approximate a physical situation where species 1 converts into species 2 by binding a third species that is part of the buffer. If the transport of this third species is fast compared with the transport of species 1 and 2, then the above equations apply. This said, we will continue to call species 1 and 2 “the monomer” and “the dimer,” respectively.

The linearity of the reaction terms in Eq. (7) renders the problem linear within an individual phase. The chemical potentials and concentrations can be presented as linear combinations of Yukawa-like terms *r*^−1^*e*^±*qr*^ while the differential equation is thus reduced to an algebraic characteristic equation for the lengths *q*^−1^ that can be solved much more readily than the original non-linear differential Eq. (2). This circumstance allows one to readily explore broad ranges of parameters. Once a non-trivial solution of the 1st order case (7) is found, one may then attempt to confirm whether a similar solution exists in the more complicated, 2nd order case from Eq. (2). Throughout, we consider exclusively the spherically symmetric geometry; such solutions are expected to minimize the surface tension between the two phases during phase coexistence^36^.

### Stationary droplet solution

We specifically inquire whether long-lived inclusions of the dimer-rich phase could form inside the monomer-rich phase, when the dimer-rich phase is in fact metastable:

8 $${\mathrm{\Delta }}g \equiv g^{({\mathrm{d}})} - g^{({\mathrm{m}})} > 0.$$

Such long-lived inclusions, if any, could represent a metastable state and/or nucleate in an activated fashion. In either case, we must look for stationary droplet-like solutions of Eqs. (3)–(7): $\dot n_i = 0$, where the minority and majority phase are the dimer-rich and monomer-rich liquids, respectively.

**Fig. 1:**
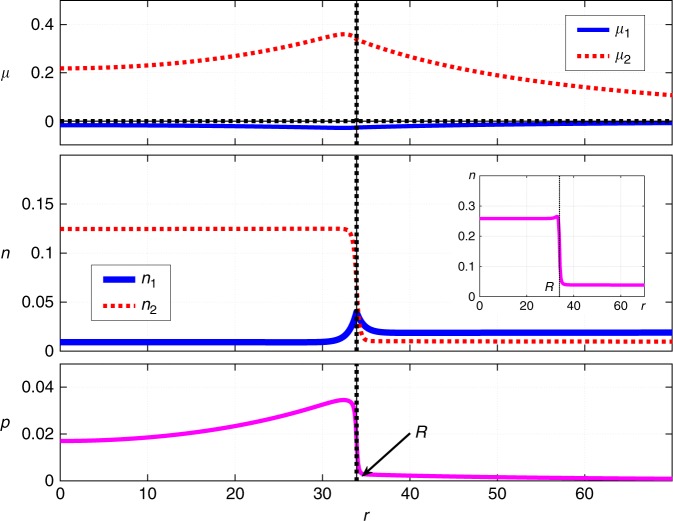
Stationary droplet solution, 1st order reaction case. Shown are the radial-coordinate dependences of the local chemical potentials *μ*_*i*_ (*i* = 1, 2), concentrations *n*_*i*_ (*i* = 1, 2), and pressure *p* for a stationary, spherically symmetric cluster of radius *R*, as obtained by solving the reaction-diffusion scheme in Eq. (7). The inset shows the *r*-dependence of the total amount of the solute, $n \equiv n_1 + 2n_2$. The following parameter values are employed: $\kappa _1^{({\mathrm{d}})} = \kappa _2^{({\mathrm{d}})} = \kappa _1^{({\mathrm{m}})} = \kappa _2^{({\mathrm{m}})} = 40$, $m_1^{({\mathrm{d}})} = m_1^{({\mathrm{m}})} = 52.36$, $m_2^{({\mathrm{d}})} = m_2^{({\mathrm{m}})} = 500$, $n_1^{({\mathrm{d}})} = 0.01$, $n_2^{({\mathrm{d}})} = 0.12$, $n_1^{({\mathrm{m}})} = 0.02$, $n_2^{({\mathrm{m}})} = 0.01$, $D_1^{({\mathrm{d}})} = 0.33$, $D_2^{({\mathrm{d}})} = 0.25$, $D_1^{({\mathrm{m}})} = 1$, $D_2^{({\mathrm{m}})} = 0.76$, $k_1^{({\mathrm{d}})} = 0.001$, $k_2^{({\mathrm{d}})} = 0.000077$, $k_1^{({\mathrm{m}})} = 0.000038$, $k_2^{({\mathrm{m}})} = 0.000077$, Δg = 0.01, $k_1^{\mathrm{\ddagger }} = 0.00005$, $k_2^{\mathrm{\ddagger }} = 0.00003$. The units are arbitrary; the unit of length can be thought of as roughly comparable to molecular dimensions and the unit of energy to *k*_*B*_*T*. The values for the rate coefficients and diffusivities were chosen to yield values for the cluster size comparable to those seen in protein solutions

**Fig. 2:**
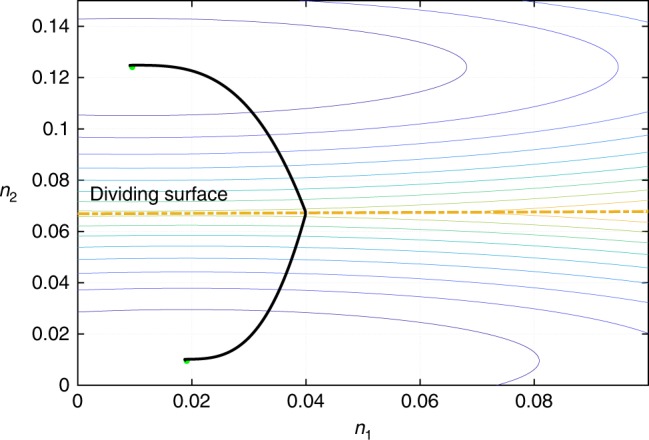
Bulk portion of the free energy. Shown is the contour plot of the presently employed bulk free energy density ${\cal{V}}(n_1,n_2)$, see Eq. (6), as a function of the concentrations *n*_1_ and *n*_2_ of the components. The two paraboloids correspond with the free energies of the two pure phases, respectively; the paraboloids intersect at the “dividing surface” shown in the plot by the dashed yelow line. The upper-left minimum corresponds to the dimer-rich solution, which is the minority phase. The curve connecting the two minima is the parametric plot of the concentrations *n*_1_ and *n*_2_ from Fig. 1, the parameter being the radial coordinate *r*

Such non-trivial stationary solutions do indeed exist as we exemplify in Fig. 1. There we show the coordinate dependences of the (local) chemical potentials and concentrations of the two species, and the hydrostatic pressure. (The coordinate-dependent pressure was computed as in ref. ^36^, see Methods.) The length *R* denotes the radius of the spherical region occupied by the dimer-rich phase. The value of *R* is determined self-consistently as a result of solving the equations. We will use *R* as the nominal cluster radius but note that it is a lower bound on the cluster size because the concentrations reach their bulk values at *r* > *R*, as should be clear from Fig. 1. In Fig. 2, we show a parametric plot of the concentrations of the monomer and dimer, the parameter being the distance from the droplet center. The parametric plot is superimposed on the contour plot of the bulk free energy ${\cal{V}}$ from Eq. (6).

As anticipated by Pan et al.^11^, the net particle exchange for each individual species, between the droplet and the bulk solution, drops exponentially fast into the bulk: The solutions *q* of the aforementioned characteristic equation are independent of the droplet radius *R*, see Methods. Now, nearer to the droplet, there is significant influx of the monomer toward the droplet and outflaw of the dimer, accompanied by a net decay of the dimer into the monomer. At the same time, the total flux of the solute, i.e., the quantity $\mathop {\sum}\nolimits_i \tilde D_i{\bf{\nabla }}\mu _i$, is identically zero in steady state.

The situation inside the droplet is drastically different from that anticipated in ref. ^11^ in that it largely mirrors the transport pattern on the outside: For the most part, the monomer flows from the center to the boundary while the dimer does the opposite. Figures 1 and 2 highlight a peculiar nature of the stationary solution at Δ*g* > 0: The chemical potentials, the concentration of the monomer, and the pressure all exhibit non-monotonic dependences on the radial coordinate *r*. In contrast, such dependences are expected to be monotonic during conventional nucleation, so as to minimize the surface tension between the minority and majority phases^36^. (Furthermore, the chemical potentials are strictly spatially uniform when the droplet is critical^26,36^!) We show separately the quantity $n \equiv n_1 + 2n_2$, which is the total concentration of the solute, irrespective of whether it is in the form of monomer or dimer. According to Fig. 1, there a small pile up of the solute at the droplet boundary.

The apparent decrease in the pressure toward the center of the droplet is expected because the pressure difference between the bulk dimer-rich and monomer-rich phases is the negative of the bulk free energy difference^36^:

9 $$p_{{\mathrm{bulk}}}^{({\mathrm{d}})} - p_{{\mathrm{bulk}}}^{({\mathrm{m}})} = - {\mathrm{\Delta }}g.$$

We have derived earlier the following expression for the pressure differential in the Landau-Ginzburg liquid free energy for a liquid mixture, in spherical geometry^36^:

10 $$p_2 - p_1 = - 2{\int}_1^2 {\frac{{dr}}{r}} \mathop {\sum}\limits_i {\kappa _i} (dn_i/dr)^2 + \mathop {\sum}\limits_i {{\int}_1^2 {n_i} } d\mu _i.$$

The first term on the r.h.s. represents the excess pressure due to the curvature of the interface and would give the venerable Laplace expression 2*σ*/*R* in the limit of a thin interface with surface tension coefficient *σ*^36^. The second term is of bulk character (i.e., independent of interface curvature) and, if in steady-state, stems exclusively from the presence of chemical conversion. According to Eq. (10), the rate at which pressure saturates, as one moves away from the interface, is determined by the largest of the aforementioned decay lengths *q*^−1^ for the spatially inhomogeneous parts of the concentrations and the chemical potential. This notion, together with Eqs. (8)–(10), implies that in a sufficiently large droplet, pressure on the inside will be lower than that in the bulk solution. This situation is in stark contrast with the common case of nucleating a stable minority phase. In the latter case, the inside pressure is always higher—consistent with the minority being the stable phase—and, furthermore, precisely matches the Laplace pressure when the nucleus is critical. Direct illustration of the latter notion can be found in ref. ^36^.

**Fig. 3:**
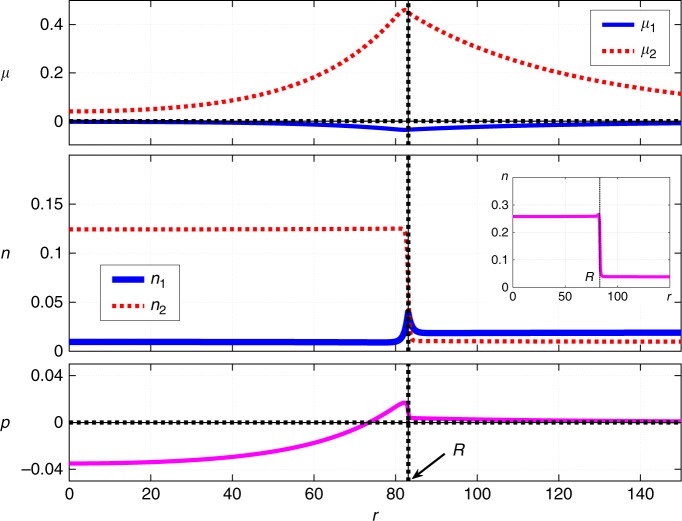
Stationary droplet solution, 1st order reaction case. The radial-coordinate dependences of the chemical potentials *μ*_*i*_ (*i* = 1, 2), concentrations *n*_*i*_ (*i* = 1, 2), and pressure *p* for a stationary cluster of radius *R*. The radial-coordinate, *r*, is measured from the center of the cluster. The inset shows the *r*-dependence of the total amount of the solute, *n* ≡ *n*_1_ + 2*n*_2_. Δ*g* = 0.04. The rest of the parameter values are the same as in Fig. 1

The finite width of the interface also implies that sufficiently large clusters can be regarded as possessing a thin interface. We note the solution shown in Fig. 1 represents a modestly-sized cluster so that the width of the cluster-bulk interface is not small compared with the cluster radius. Thus, although the pressure does show a trend toward lower values deeper into the droplet, it does not reach its bulk value for the minority phase even at *r* = 0. In Fig. 3, we exemplify a contrasting solution for a sufficiently large droplet such that the quantity ${\mathrm{\Delta }}p \equiv p(r = 0) - p(r = \infty )$ is indeed numerically close to −Δ*g*.

### Droplet growth and decay, and the ripening of the cluster ensemble

**Fig. 4:**
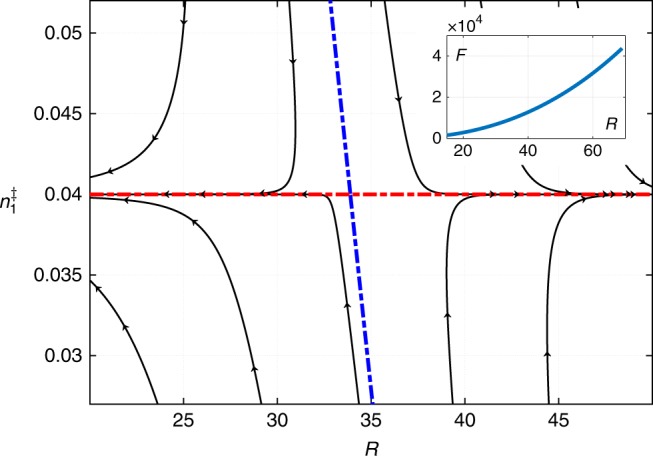
The flow chart for the cluster size *R* and composition $n_1^{\mathrm{\ddagger }}$ of the monomer at the boundary. The blue dashed separates the regions where the droplet is subcritical (l.h.s.) and supercritical (r.h.s.), respectively. The dashed red line shows the stationary values of the $n_1^{\mathrm{\ddagger }}$ for all values of *R*. The stationary solution in terms of both variables is unique and is located at the intersection of the blue and red dashed lines. The parameter values are the same as in Fig. 1

We next ask whether the above droplet solution represents a metastable entity or a transition state configuration. To answer this question, we do not attempt to solve the full-blown time dependent problem. Instead, we first artificially constrain the values of the concentrations at the droplet boundary and the droplet radius *R* away from their stationary values. We then use the resulting profiles of the chemical potentials to determine the fluxes of the monomer and dimer at the cluster boundary. In turn, these fluxes are used to estimate the value of the time derivatives $\dot R$ and $\dot n_1^{\mathrm{\ddagger }}$, where $n_1^{\mathrm{\ddagger }}$ is the concentration of the monomer at the boundary; this is detailed in the Methods. ($n_2^{\mathrm{\ddagger }}$ is specified automatically because the boundary is a line in the $(n_1^{\mathrm{\ddagger }},n_2^{\mathrm{\ddagger }})$ plane.) Finally, we make a flow chart corresponding to the vector $(\dot R,\dot n_1^{\mathrm{\ddagger }})$ in the $(R,n_1^{\mathrm{\ddagger }})$ plane, as shown in Fig. 4. This flow chart demonstrates that the stationary solution is, in fact, a critical point beyond which the droplet will grow indefinitely but evaporate otherwise. At the same time, we note the free energy of the droplet is a monotonically increasing function of the droplet radius, as we show in the inset of Fig. 4. When combined, these two notions would seem to indicate the droplet will grow indefinitely despite its free energy increasing in the process. This would seem to contradict the second law of thermodynamics.

This situation is, however, simply a formal consequence of the present treatment being incomplete as it does not account for thermal noise, see the remark following Eq. (2). In actuality, thermal noise will eventually cause the droplet to decay toward the stable, bulk phase and in fact do so in an activated fashion. Indeed, we first observe that similarly to conventional nucleation, the presently found droplet solutions exhibit a well defined interface tension. This could be seen from the rapid onset of the “Laplace”pressure due to the interface curvature, see Figs. 1 and 3, and the first term on the r.h.s. of Eq. (10). Because of this interface tension, the droplet will be stable against modest fluctuations in shape. Consequently, the droplet’s decay will be subject to a free energy barrier.

We have not succeeded in finding spherically symmetric stationary solutions for the problem of reverse nucleation, i.e., the nucleation of the bulk phase inside the minority phase in situations where the forward nucleation of the minority phase does exhibit such stationary solutions. If such reverse solutions do not exist—something we can not demonstrate with complete certainty at present—the situation is the opposite to that arising during regular nucleation. In the latter case, spherically symmetric stationary solutions exist for nucleating a stable minority phase but not the other way around.

**Fig. 5:**
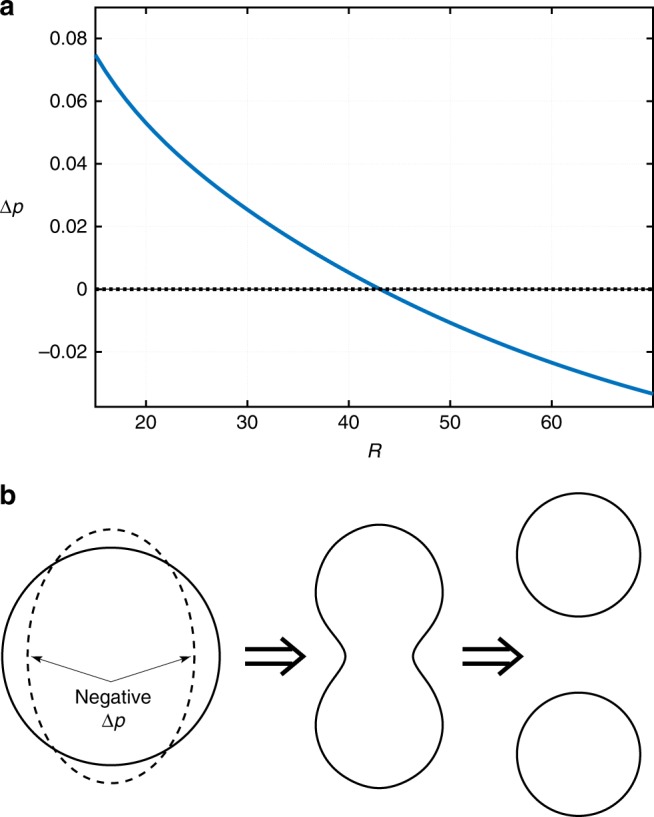
Decrease in the internal pressure with the cluster size and the resulting mechanical instability. **a** The solid line shows the pressure differential ${\mathrm{\Delta }}p \equiv p(r = 0) - p(r = \infty )$ between the cluster centre and the solution bulk, as a function of the droplet radius. Only one point corresponds to a stationary solution, which is the same as that in Fig. 4. The Δ*p* < 0 region, below the dashed line, corresponds to mechanically unstable configurations. **b** A graphical explanation of the mechanical instability and subsequent breaking of a droplet as the pressure differential becomes negative. The flatter portions of the interface will cave first

Thus the activated droplet decay must involve non-spherically symmetric geometries as its transition state configurations. A clue as to how such geometries could arise can be obtained by plotting the (local) pressure in the center of the sphere relative to its value in the solution bulk, ${\mathrm{\Delta }}p \equiv p(r = 0) - p(r = \infty )$, as a function of the droplet radius *R* for a spherical droplet, see Fig. 5a. Clearly, the pressure differential between the inside and outside of the droplet becomes negative for a sufficiently large droplet potentially signaling a mechanical instability. This is particularly straightforward to see in the limit of a thin interface. Hereby, the pressure inside the droplet quickly reaches the bulk value for the minority phase already right under the surface of the droplet. Under these circumstances, the metastability of the minority phase implies, by Eq. (9), that the pressure on the inside is less than on the outside. In turn, this means the droplet is mechanically unstable. Indeed, because of shape fluctuations, some parts of the boundary will acquire curvature less than 2/*R*. The local pressure in the adjacent regions, inside the droplet, will become negative, relative to the bulk, leading to a caving of the interface. In turn, this will lead to a further decrease in (the Laplace contribution to) the inner pressure, by Eq. (10), and so on. The interface will thus buckle leading to a necking of the cluster; this is illustrated in Fig. 5b.

The interface is however not thin. For instance, the configuration shown in Fig. 1 exhibits an interface whose width is a substantial fraction of the cluster radius. Under such circumstances, the pressure on the inside does not reach its bulk value for the minority phase even in the center of the droplet. In addition, the said buckling instability would be further offset for thick slabs^38^. Thus we conclude that the droplet radius *R*_max_ beyond which ${\mathrm{\Delta }}p \equiv p(r = 0) - p(r = \infty ) < 0$ likely represents a lower bound on the size where the mechanical instability sets in.

The following microscopic picture thus emerges: In a steady-state solution, clusters continuously nucleate, grow, and ultimately decay because of a mechanical instability. The latter instability ultimately stems from the dimer-rich phase being thermodynamically metastable. For each nucleating cluster, there is a decaying one, in steady-state, and so there is no net entropy production or consumption. The monotonic increase of the free energy of an individual droplet with the droplet size drives home the notion that the clusters are stabilized kinetically, not thermodynamically. The stabilization comes about because once formed, as a result of density fluctuations, a dimer-rich region will extend at least for the distance dimers will typically travel before they decay back into monomers.

**Fig. 6:**
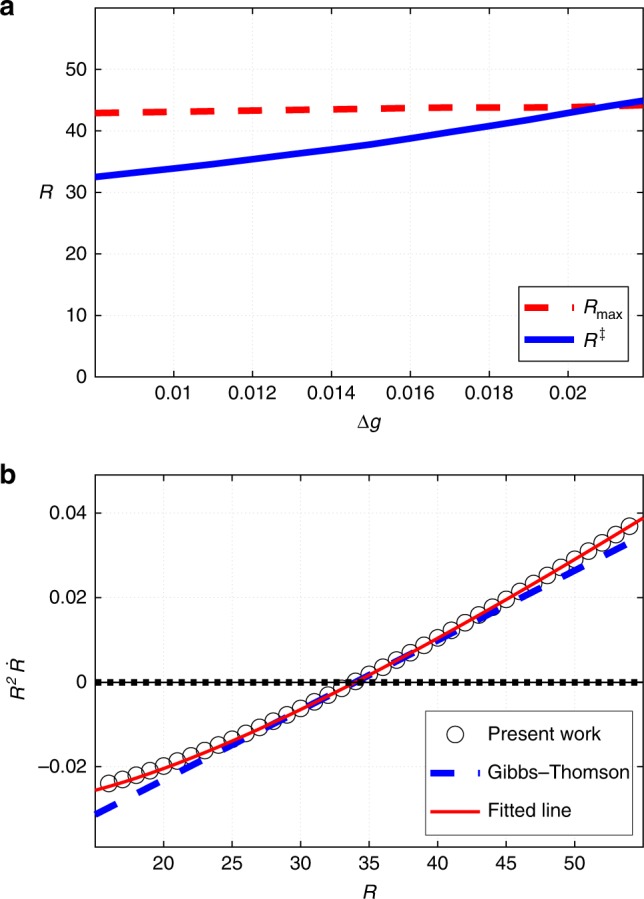
Size range and the growth rate of clusters. **a** The Δ*g* dependences of the critical radius *R*^‡^ and the threshold value of the cluster *R*_max_ beyond which the cluster becomes mechanically unstable. **b** The dependence of the rate of volumetric growth of the cluster, as reflected in the quantity $R^2\dot R$, is shown as a function of the cluster’s radius. The present data are shown with circles, the corresponding smooth fit with the thin red line. This is contrasted with with the prediction for the traditional case of nucleation of a stable minority phase, due to Gibbs–Thomson, shown using the dashed blue line. The slope of the latter is chosen to match the present results. Only one point on the curves corresponds to a stationary solution, as in Fig. 5a

One may further elaborate on the above notions of kinetic stabilization. The reaction terms in Eqs. (2) and (7) are local and thus the kinetic stabilization, if any, would be of bulk character. On the other hand, such stabilization can operate only on lengths not exceeding the kinetic lengths of the type in Eq. (1). Thus we conclude that for the present scenario to be viable, the parameter values should be such that the critical size *R*^‡^ for nucleation is less than the pertinent kinetic length. We can check this notion, even if somewhat indirectly, by computing the critical size for a range of Δ*g* values. Larger values of Δ*g* should imply less overall stabilization—thermodynamic plus kinetic—and, consequently, larger values for the critical radius. This is borne out by the results in Fig. 6a. In that Figure, we also show the dependence of the threshold value of the droplet radius *R*_max_ at which the pressure differential Δ*p* in the center of the droplet would vanish. We observe that, indeed, there is an upper limit on the bulk free energy excess of the dimer-rich phase beyond which already sub-critical clusters would be mechanically unstable and, thus, could not emerge in the first place. We reiterate that *R*_max_ shown in Fig. 6a is likely a lower bound on the actual size where the mechanical instability sets in. Because the characteristic equations are complicated, it is difficult to see the explicit dependence of the lengths in Fig. 6a on the kinetic coefficients. We have checked that for specific values of parameters, the critical radius *R*^‡^ does decrease with the decay rate *k*_2_ of the dimer, consistent with the heuristic arguments of Pan et al.^11^; the corresponding data can be found in Methods.

Figure 6a indicates the range of possible values for the cluster size. Indeed, because sub-critical clusters would rapidly evaporate, one should readily observe clusters only within the size range specified by the critical size *R*^‡^ and the threshold size *R*_max_ (if $R^{\mathrm{\ddagger }} < R_{{\mathrm{max}}}$). According to Fig. 6a, this range is relatively modest, consistent with the apparent weak dependence of the cluster size on the concentration of the solute.

Next, we address the question of the ripening of the clusters in a freshly prepared solution. According to refs. ^24–27^, conventional Ostwald ripening comes about for the following reason: If the typical size of the minority phase is sub-macroscopic, the solution of the pertinent species in the majority phase is over-saturated, the degree of supersaturation decreasing with the typical droplet size according to the usual Gibbs–Thomson relation^7^. At a given value of supersaturation, droplets smaller than the corresponding critical size will evaporate, while droplets that are bigger than the critical size will grow. As a result, the average droplet size will grow until the minority phase reaches macroscopic dimensions while the supersaturation peters out. Within the Gibbs–Thomson approximation and in the limit of diffusion controlled droplet growth, the volumetric rate of droplet growth, $R^2\dot R$ happens to scale linearly with the deviation $(R - R^{\mathrm{\ddagger }})$ of the droplet radius from the critical radius, see Methods. This is shown by the dashed line in Fig. 6b. The corresponding dependence for the present clusters is shown in that Figure with symbols. Although different from a strict linear form, the quantity $R^2\dot R$ for kinetically stabilized clusters is still a monotonically increasing function of *R* vanishing at $R = R^{\mathrm{\ddagger }}$. As detailed in Methods, the data in Fig. 6b imply that well before the steady-state cluster is reached, clusters grow according to a power-law $R^{\mathrm{\ddagger }} \propto = t^{0.32 \pm 0.01}$. This is quite close to if not somewhat faster than the dependence *t*^0.26±0.03^ observed by Ye Li et al.^12^. For comparison, the Lifshitz-Slyozov-Wagner^24,25^ (LSW) mechanism of conventional Oswald ripening predicts $R^{\mathrm{\ddagger }} \propto = t^{1/3}$.

This notion suggests that an Ostwald-like ripening could take place in cluster-containing solutions. Indeed, according to Fig. 6a, the critical radius increases with Δ*g*, as already mentioned. On the other hand, Δ*g* increases with lowering of the concentration of the solute in the bulk solution $\left( {g^{(1)} \sim {\mathrm{ln}}\,n_{1,b}} \right)$. In a freshly prepared solution, the typical cluster size is less than its value in equilibrium, resulting in an excess solute to compensate for the excess curvature of the cluster surface. As the average cluster size increases, the amount of this excess solute will decrease leading to an increase in Δ*g*, and, consequently an increase in the critical radius. The increase of the critical radius with time is similar to what happens during conventional Ostwald ripening. In contrast with the conventional Ostwald ripening, however, the supersaturation due to the finite curvature increases, not decreases with time. This is because the minority phase here is thermodynamically metastable in the first place. Yet as in the case of growth of an individual droplet, the seemingly “positive-feedback loop” for Δ*g* does not lead to a runaway growth of the droplets because of the mechanical instability discussed earlier. Furthermore, as Δ*g* approaches its limiting value, where $R^{\mathrm{\ddagger }} = R_{{\mathrm{max}}}$, the time dependence of the typical cluster size must stop following the Ostwald-like *t*^1/3^ and, instead, level off at the equilibrium value of *R*_max_.

It is interesting to ask how long it would take the system to reach this stationary state. Existing experimental studies of cluster ripening in lysozyme solutions^12^ indicate ripening times of the order hours, while noting that the LSW treatment would predict seconds. Consistent with this (hitherto unexplained) discrepancy, the presently predicted scaling of the time dependence of the cluster radius the detailed $\dot R$ vs. *R* dependence is quite distinct from that on which the LSW theory is predicated. In the absence of actual treatment, which is work in progress, we provide here only a qualitative estimate using the LSW formalism. Hereby, one may linearize the $R^2\dot R$ dependence on *R* in Fig. 6b, near the critical value of the latter, to connect the rate of cluster growth with its radius, see Methods. In agreement with conclusions of ref. ^12^, we obtain that the LSW-based time needed to reach the size *R*_max_ is about 2 s, for the parameter values in Fig. 6 and the diffusivity of lysozyme molecule reported in ref. ^11^.

Finally, we note that there may be additional processes present within the dimer-rich phase, such as gelation seen in lysozyme-rich liquid, both macroscopic^39^ and mesoscopic^17^. Such additional processes may further stabilize the clusters.

### The second-order case

**Fig. 7:**
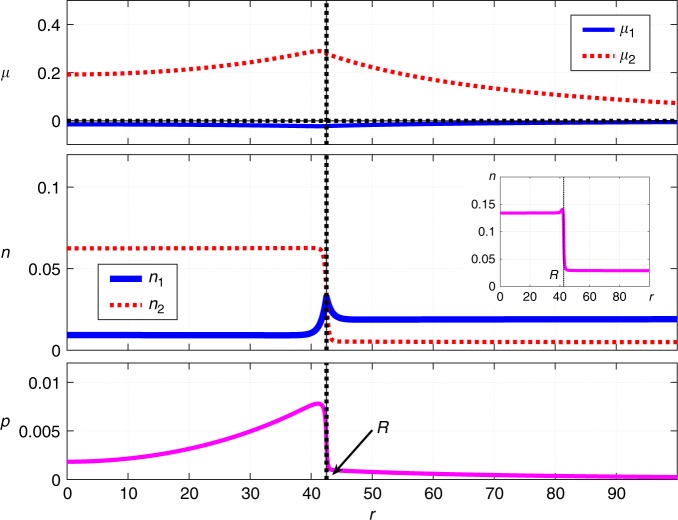
Stationary droplet solution, 2nd order reaction case. Shown are the radial-coordinate dependences of the chemical potentials *μ*_*i*_ (*i* = 1, 2), concentrations *n*_*i*_ (*i* = 1, 2), and pressure *p* for a stationary, spherically symmetric cluster of radius *R*, as obtained by solving the reaction-diffusion scheme in Eq. (2). The inset shows the *r*-dependence of the total amount of the solute, $n \equiv n_1 + 2n_2$. The following parameter values are employed: $\kappa _1^{({\mathrm{d}})} = \kappa _2^{({\mathrm{d}})} = \kappa _1^{({\mathrm{m}})} = \kappa _2^{({\mathrm{m}})} = 40$, $m_1^{({\mathrm{d}})} = m_1^{({\mathrm{m}})} = 52.36$, $m_2^{({\mathrm{d}})} = m_2^{({\mathrm{m}})} = 500$, $n_1^{({\mathrm{d}})} = 0.0095$, $n_2^{({\mathrm{d}})} = 0.062$, $n_1^{({\mathrm{m}})} = 0.019$, $n_2^{({\mathrm{m}})} = 0.005$, $D_1^{({\mathrm{d}})} = 0.33$, $D_2^{({\mathrm{d}})} = 0.13$, $D_1^{({\mathrm{m}})} = 1$, $D_2^{({\mathrm{m}})} = 0.38$, $k_1^{({\mathrm{d}})} = 0.026$, $k_2^{({\mathrm{d}})} = 0.000019$, $k_1^{({\mathrm{m}})} = 0.0005$, $k_2^{({\mathrm{m}})} = 0.000019$, Δ*g* = 0.01

Here we present, in Fig. 7, the stationary solution corresponding to the original second-order reaction setup from Eq. (2). This solution was obtained using the finite element method^40^ and requires much more effort than the first-order case, both in terms of implementation and computation proper; see Methods for details. Note that both in the 1st order and 2nd order kinetics case, we assumed that the decay rate of the dimer has the same value in both phases. This is to reflect the expectation that, in contrast with the binding rate for the monomer, the decay rate of the complex would not be very sensitive to the composition of the solution. We have obtained stationary solutions for other values of the parameters as well. In any event, we observe that the non-linearity in the reaction terms does not destroy the kinetic stabilization observed in the case of first order kinetics; the two cases produce qualitatively similar results. At the same time, we note introducing the non-linearity in the reaction kinetics does have substantial quantitative effects. For instance, for the same values of the parameters that yield a droplet solution when the complexation reaction is second order, the corresponding linearized case may not exhibit a droplet solution altogether, steady-state or not.

## Methods

### Boundary conditions and the non-stationary case

To describe phase coexistence we employ a double-minimum bulk free energy density ${\cal{V}}\left( {n_1,n_2} \right)$. The latter free energy corresponds with the grand-canonical ensemble and is straightforwardly related to the Helmholtz free energy density *f*^36^:

11 $${\cal{V}}\left( {n_1,n_2} \right) = f\left( {n_1,n_2} \right) - \mu _{1,b}n_1 - \mu _{2,b}n_2.$$

where *μ*_*i*,*b*_ is the chemical potential of species *i* in the bulk.

A smooth surface exhibiting two minima has to be a quartic polynomial or a more complicated function, which renders even the otherwise linear differential Eq. (7) highly non-linear and difficult to solve even numerically. To circumvent this difficulty, we employ a bulk free energy which is not smooth but, instead, consists of two intersecting paraboloids, see Eq. (6) and Fig. 2. The resulting free energy surface exhibits a singularity, in the form of a discontinuous gradient, where the two paraboloids from Eq. (6), *α* = m and *α* = d, intersect. The singularity is however confined to a region of measure zero, the latter region corresponding to the phase boundary. In each individual phase, the transport part of the problem reduces to linear differential equations. The respective solutions must be patched together where the bulk free energy is singular, i.e., at the phase boundary. Patching such solutions for mixtures, as opposed to systems described by only one order parameter, presents some subtlety and has been worked out earlier by us^36^.

12 $$n_i(R^ + ) = n_i(R^ - ) \equiv n_i^{\mathrm{\ddagger }}$$

13 $$\mu _i(R^ - ) = \mu _i(R^ + )$$

14 $$\mathop {\sum}\limits_i \left. {\kappa _i(\partial n_i/\partial r)^2} \right|_{R^ - }^{R^ + } = 0.$$

While Eqs. (12) and (13) are intuitive, the constraint in Eq. (14) is less obvious and comes about because the hydrostatic pressure is continuous across the boundary:

15 $$\left. {p(r)} \right|_{R^ - }^{R^ + } = 0.$$

The pressure for the Landau-Ginzburg functional is computed according to^36^:

16 $$p(r) = - {\cal{V}} + \mathop {\sum}\limits_i {\mu _i} n_i + \mathop {\sum}\limits_i {\frac{{\kappa _i}}{2}} \left( {\frac{{dn_i}}{{dr}}} \right)^2.$$

In the stationary case, $\dot R = 0$, the fluxes for each component must be continuous as well:

17 $$\left. {\left( {\tilde D_i\frac{{\partial \mu _i}}{{\partial r}}} \right)} \right|_{R^ - }^{R^ + } = 0$$

Note we must separately specify the reaction rates at the boundary, which we denote with $k_i^{\mathrm{\ddagger }}$. For the 1st order reaction case, the kinetic equations at the boundary read

18 $$\dot n_1^{\mathrm{\ddagger }} = - k_1^{\mathrm{\ddagger }}n_1^{\mathrm{\ddagger }} + k_2^{\mathrm{\ddagger }}n_2^{\mathrm{\ddagger }}$$

19 $$\dot n_2^{\mathrm{\ddagger }} = k_1^{\mathrm{\ddagger }}n_1^{\mathrm{\ddagger }} - k_2^{\mathrm{\ddagger }}n_2^{\mathrm{\ddagger }}$$

and analogously for more complicated reaction schemes.

The above equations form a complete set of equations that allow one to determine, self-consistently, the stationary value of the droplet radius *R*. This setup is over-defined in the sense that not all parameters are independent. Clearly, the reaction rates and equilibrium concentrations are not independent:

20 $$k_1^{(\alpha )}(n_{1,b}^{(\alpha )})^2 = 2k_2^{(\alpha )}n_{2,b}^{(\alpha )}$$

for the 2nd order reaction and for the 1st order case:

21 $$k_1n_{1,b}^{(\alpha )} = k_2n_{2,b}^{(\alpha )}.$$

Here *α* labels the phase. A more subtle constraint on the parameters comes about because of particle conservation. Indeed, adding together the two equations in Eq. (7) and using Eq. (3) yields

22 $$\nabla ^2(\tilde D_1\mu _1 + \tilde D_2\mu _2) = 0$$

In equilibrium, this equation is solved by by $\tilde D_1\mu _1 + \tilde D_2\mu _2 = 0$, since $\mu _i(r = \infty ) = 0$ by construction. Thus one obtains

23 $$\tilde D_1\mu _1 = - \tilde D_2\mu _2.$$

Combining this with the boundary condition (13) yields

24 $$\tilde D_1^{({\mathrm{m}})}/\tilde D_2^{({\mathrm{m}})} = \tilde D_1^{({\mathrm{d}})}/\tilde D_2^{({\mathrm{d}})}.$$

It is straightforward to see that the same constraint must be satisfied in the 2nd-order kinetics case.

To approach non-stationary situations, we make additional assumptions. First, we specify for concreteness that a droplet of the minority phase be a vapor bubble with respect to the monomer, but a liquid droplet with respect to the complex:

25 $$\begin{array}{*{20}{l}} {n_{1,b}^{({\mathrm{d}})}} \hfill & < \hfill & {n_{1,b}^{({\mathrm{m}})}} \hfill \\ {n_{2,b}^{({\mathrm{d}})}} \hfill & > \hfill & {n_{2,b}^{({\mathrm{m}})},} \hfill \end{array}$$

while assuming the monomer is the primary species in the majority phase:

26 $$n_{1,b}^{({\mathrm{m}})} > n_{2,b}^{({\mathrm{m}})}.$$

Next we make the usual approximation^26^ by which the interface is assumed to move on timescales that are much longer than the diffusion times scales *R*^2^/*D*. (*D* is the regular diffusivity, see below.) And so for each value of *R*, we solve the stationary equations $\dot n_i^{\mathrm{\ddagger }} = 0$ while relaxing the constraint (17) that the fluxes of the components on the opposite sides of the boundary be equal. Using these assumptions, we (approximately) infer the sign of the rate of change of the droplet radius away from steady state^26^:

27 $$\begin{array}{*{20}{l}} {\dot R} \hfill & \approx \hfill & {\frac{{ - 1}}{{n_{1,b}^{({\mathrm{m}})} - n_{1,b}^{({\mathrm{d}})}}}\left. {\left( {\tilde D_1\frac{{\partial \mu _1}}{{\partial r}}} \right)} \right|_{R^ - }^{R^ + }} \hfill \\ {} \hfill & = \hfill & {\frac{{ - 1}}{{n_{2,b}^{({\mathrm{m}})} - n_{2,b}^{({\mathrm{d}})}}}\left. {\left( {\tilde D_2\frac{{\partial \mu _2}}{{\partial r}}} \right)} \right|_{R^ - }^{R^ + }} \hfill \end{array}$$

Note Eq. (27) represents an additional constraint. Thus pegging *R* and $n_1^{\mathrm{\dagger }}$ away from their stationary values allows one to find self-consistently to determine the values of $\dot R$ and, for instance, $\dot n_1^{\mathrm{\dagger }}$. The corresponding flow chart is shown as Fig. 4 and demonstrates that the stationary solution in fact represents a transition state, not a metastable configuration.

### First order reaction

When the monomer-dimer conversion is a first order reaction, the problem reduces to a set of two linear, fourth-order differential equations, for each individual phase:

28 $$\begin{array}{*{20}{l}} {\dot n_1} \hfill & = \hfill & {\tilde D_1\nabla ^2( - \kappa _1\nabla ^2n_1 + m_1n_1) - k_1n_1 + k_2n_2,} \hfill \\ {\dot n_2} \hfill & = \hfill & {\tilde D_2\nabla ^2( - \kappa _2\nabla ^2n_2 + m_2n_2) + k_1n_1 - k_2n_2.} \hfill \end{array}$$

subject to the the patching conditions discussed above and the boundary conditions in the center of the droplet, *r* = 0, and in the bulk, *r* = ∞. In a standard fashion, we require that

29 $$\begin{array}{*{20}{l}} {n_i(r = \infty )} \hfill & = \hfill & {n_{i,b}} \hfill \\ {{\bf{\nabla }}n_i(r = 0)} \hfill & = \hfill & {0.} \hfill \end{array}$$

and

30 $$\begin{array}{*{20}{l}} {\mu _i(r = \infty )} \hfill & = \hfill & 0 \hfill \\ {{\bf{\nabla }}\mu _i(r = 0)} \hfill & = \hfill & {0.} \hfill \end{array}$$

**Fig. 8:**
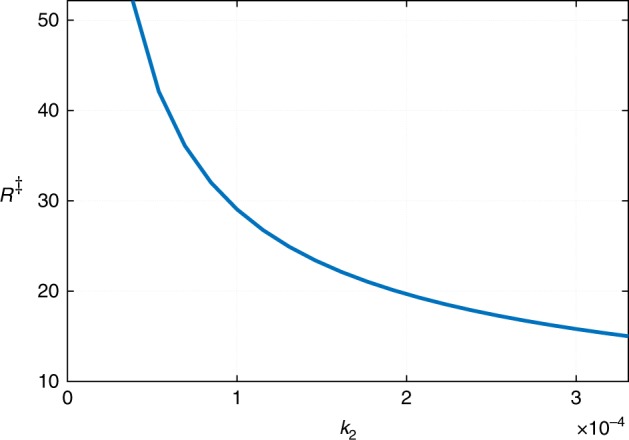
Dependence of the critical radius on the decay rate of the dimer $k_2 \equiv k_2^{({\mathrm{d}})} = k_2^{({\mathrm{m}})}$. The rest of the parameter values are the same as in Fig. 1

The linear Eq. (28) are solved by a linear superposition of Yukawa potential-like functions *e*^*qr*^/*r*^36^. The characteristic equation for the wavevector *q* can be written in a relatively transparent form:

31 $$\begin{array}{*{20}{l}} 0 \hfill & = \hfill & {q^6 - q^4\left( {l_1^{ - 2} + l_2^{ - 2}} \right)} \hfill \\ {} \hfill & {} \hfill & { + q^2\left[ {\left( {l_1l_2} \right)^{ - 2} + \left( {l_1L_1} \right)^{ - 2} + \left( {l_2L_2} \right)^{ - 2}} \right]} \hfill \\ {} \hfill & {} \hfill & { - \left[ {\left( {l_1l_2L_1} \right)^{ - 2} + \left( {l_1l_2L_2} \right)^{ - 2}} \right],} \hfill \end{array}$$

where $l_i^2 \equiv \kappa _i/m_i$ and $L_i^2 = D_i/k_i$. Here,

32 $$D_i \equiv \tilde D_im_i$$

is the ordinary diffusivity. Indeed, Eqs. (5) and (3) together with the usual ${\mathbf{j}}_i = - D_i{\bf{\nabla }}n_i$ lead to Eq. (32). The lengths *l*_*i*_ are, of course, the correlation lengths of the Landau-Ginzburg theory^32^; they are static, thermodynamic quantities. In contrast, the lengths *L*_*i*_ originate exclusively from the presence of chemical conversion and are kinetic quantities that constitute new length-scales in the problem analogously to the length scale from Eq. (1). We observe that according to Fig. 8, the critical radius is largely determined by those kinetic lengths.

The contributions of the respective terms *e*^*qr*^/*r* to the overall solution of the differential equations are constrained by the boundary and patching conditions, in the usual way^36^. Cases when the characteristic roots are degenerate can be dealt with straightforwardly. For instance, the doubly degenerate root *q* = 0 corresponds, respectively, to an additive constant and a 1/*r* contribution to the overall solution.

### Second-order reaction

As before, we solve exclusively for the stationary state within each individual phase. The stationary non-linear equations are solved using the finite differences^40^. We sub-divide the space into three spherically symmetric regions, all centered at the origin: (1) the minority phase, *r* < *R*, (2) the vicinity of the cluster in the majority phase, *R* < *r* < *R*_*p*_, and (3) the outer regions, *r* > *R*_*p*_. The edge of the outer region, *R*_*p*_, is chosen to be sufficiently far away from the cluster boundary so that the concentrations of the components are numerically close to their bulk values. Thus in the outer region, the reaction-diffusion scheme can be approximated by linearized equations in a controlled fashion:

33 $$\begin{array}{*{20}{l}} {\dot n_1} \hfill & = \hfill & { - {\bf{\nabla }}{\mathbf{j}}_1 - 2k_1\delta n_1n_{1,b} + 2k_2\delta n_2} \hfill \\ {\dot n_2} \hfill & = \hfill & { - {\bf{\nabla }}{\mathbf{j}}_2 + k_1\delta n_1n_{1,b} - k_2\delta n_2.} \hfill \end{array}$$

where $\delta n_i \equiv n_i - n_{i,b}$ is the deviation of concentration of species *i* from its bulk value. The solution of the linearized Eq. (33) is obtained exactly the same way as the first order case from Eq. (7).

In regions 1 and 2, we solve the original non-linear equation using finite differences while imposing patching conditions with the linearized solution in region 3, at *R* = *R*_*p*_. The patching is done by enforcing that the density and the chemical potential of both species be continuously differentiable at *r* = *R*_*p*_. The boundary conditions at the cluster center, *r* = 0, at the phase boundary, *r* = *R*, and in the bulk, *r* = ∞ are the same as in the 1st order case.

**Fig. 9:**
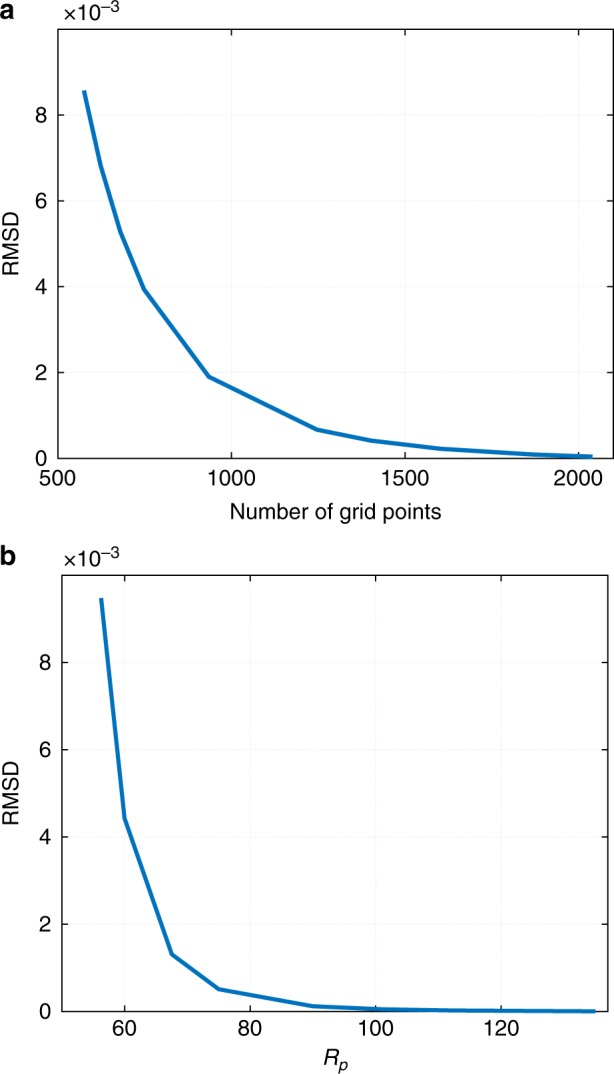
2nd-order reaction case. **a** The root-mean-square deviation of *μ*_1_ from a reference solution as a function of the number of grid points. The number of grid points of the reference solution is 2800. *R*_*p*_ = 150. **b** The root-mean-square deviation of *μ*_1_ from a reference solution as a function of the patching radius *R*_*p*_. In that reference solution, *R*_*p*_ = 150. The number of grid points is fixed at 26 per unit length. The rest of the parameter values are the same as in Fig. 7

To test the convergence of our solutions, we have computed them at several values of the grid size and the patching radius *R*_*p*_. We then evaluated the root-mean-square (RMS) difference between these solutions and the reference solution, which was obtained using some large values for the number of grid points and *R*_*p*_ respectively. In Fig. 9a, b, we show the respective RMS differences for the chemical potential of the monomer. These graphs indicate that our solutions do in fact tend to a stationary value as the number of grid points and *R*_*p*_ are increased.

### Ripening

Consider regular Ostwald ripening. At a given value of over-saturation Δ of the majority phase, the rate of growth of an individual droplets is given by^24^:

34 $$\dot R = \frac{D}{R}\left( {{\mathrm{\Delta }} - \frac{{\alpha _0}}{R}} \right),$$

where *D* is the diffusivity of the species in question and the coefficient *α*_0_ is proportional to the mismatch penalty between the majority and minority phases^7^. The critical radius is thus given by

35 $$R^{\mathrm{\ddagger }} = \alpha _0/{\mathrm{\Delta }}.$$

Eq. (34) can be profitably rewritten in terms of the critical radius and the dimensionless radius $\tilde R \equiv R/R^{\mathrm{\ddagger }}$:

36 $$\frac{{d\tilde R}}{{dt}} = \frac{{\alpha _0D}}{{R^{{\mathrm{\ddagger }}^{\mathrm{3}}}}}\left( {1 - \frac{1}{{\tilde R}}} \right)\frac{1}{{\tilde R}}$$

Lifshitz and Slyozov^24^ have argued that at sufficiently long times, the droplet size distribution tends toward a universal form that is determined by the critical radius *R*^‡^ alone and no other length scales. In other words, the distribution of the dimensionless radius $\tilde R$ is time independent at long times. Averaging Eq. (36) w.r.t. to this distribution immediately shows that for this equation to be internally consistent, one must have at long times:

37 $$R^{\mathrm{\ddagger }} = c(D\alpha _0t)^{1/3},$$

where *c* is a numerical constant of order one. (The constant turns out to be 2/9 in the simplest treatment^24^). To avoid confusion, we note that the times are sufficiently long that the memory of the initial distribution of the droplet sizes is already lost but not too long so that the number of clusters is still sub-thermodynamic. Eq. (34) implies that the volumetric rate of droplet growth is linear in the quantity $R - R^{\mathrm{\ddagger }}$:

38 $$R^2\dot R \propto (R/R^{\mathrm{\ddagger }} - 1).$$

According to the discussion in the main text, our kinetically stabilized clusters will exhibit ripening. Since they do not obey the exact linear relation (38) we may inquire whether the ripening exponent in the *R*^‡^ vs. *t* relation would differ significantly from the value 1/3 from Eq. (37) predicted by the Lifshitz-Slyozov-Wagner theory and, in the first place, from the experimental data due to Li et al.^12^. To answer this question, we first fit the pertinent curve in Fig. 6 by a functional form:

39 $$R^2\dot R \propto R^xR^{{\mathrm{\ddagger }}^z}(R^y - R^{{\mathrm{\ddagger }}^y})$$

Hereby, the Gibbs–Thomson relation and diffusion-limited droplet growth would correspond to *x* = 0, *y* = 1, and *z* = −1.) The same line of logic that led to Eq. (37) yields

40 $$R^{\mathrm{\ddagger }} \propto t^{1/[3 - (x + y + z)]}$$

**Fig. 10:**
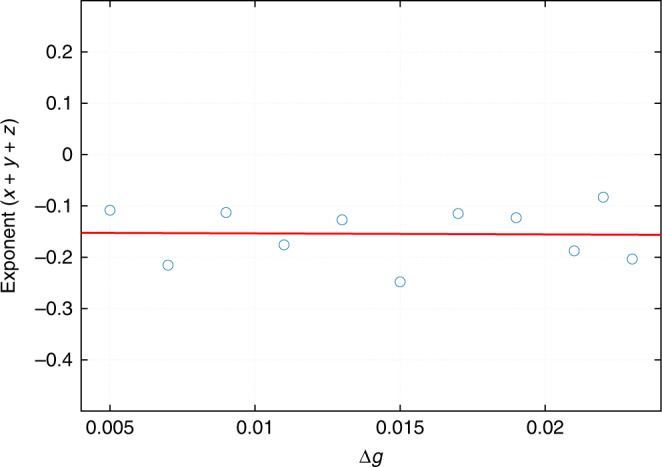
Quantification of cluster ripening. The Δ*g* dependence of the combination (*x* + *y* + *z*) of the parameters from Eqs. (39) and (40). The two equations provide, respectively, a fitting form for the size dependence of the volumetric growth rate and the corresponding scaling of the typical cluster size with time, at large values of the latter. Δ*g* is the bulk free energy excess of the minority phase per unit volume from Eq. (8). The parameter values are the same as in Fig. 1

In Fig. 10, we show the Δ*g* dependence of the combination $(x + y + z)$ of the parameters from Eqs. (39) and (40). We observe that by Eq. (40), the predicted growth implies $R^{\mathrm{\ddagger }} \propto t^{1/(3.1 \pm 0.1)} = t^{0.32 \pm 0.01}$, which is quite close to both the experiment by Ye Li et al.^12^ and the predictions due to the Lifshitz-Slyozov-Wagner theory^24–27^. We note that we have not shown that the shape of the cluster-size distribution is, in fact, time-invariant within the present framework, which would be necessary to fully validate Eq. (39). This is work in progress. Still, experimental data due to Ye Li et al.^12^. suggest that the distribution’s shape is, in fact, time-invariant.

To obtain the estimate for the cluster-ripening times cited in the main text, we set the LSW-based *c* = 2/9 in Eq. (37) and $R^{\mathrm{\ddagger }} = R_{{\mathrm{max}}}$. The quantity *α*_0_*D* is determined by fitting the curve in Fig. 6b using the functional form (38) while the diffusivity is set at the value reported for lysozyme in ref. ^12^.

## Data Availability

The reported numerical data were obtained by numerical solution of algebraic and differential equations, as detailed in the article, using the commercially available mathematical software Matlab. When appropriate, the convergence of the solutions is described. All generated data are presented in the published article.
